# Women in Ohio Medical Society

**Published:** 1875-08

**Authors:** 


Ohio State Medical Society.—* * * Alas! already at this writing, only half an hour after the opening of the session, a bomb-shell has burst amongst us. Dr. ——, of ——, has just proposed for membership *Mrs.* Dr. Glenn, of Van Wert, O., and *Mrs.* Dr. Helen Betts, of Napoleon, O., and the promise of work fades like a dream. The two ladies sit just before me as I write, and await the action of the convention with that patience peculiar to the sex. Theirs would be both very pleasant faces in the sick room; brown eyes has one, with rippling auburn hair; blue eyes the other, whose hair is freely sprinkled with gray. We do not trust ourselves to write much in the way of further personal description, though the temptation is very strong.

Some member at this juncture moves to refer these nominations to the proper committee, which defers action for the present. * * *

A number of papers and reports were now called, but there was no response or a petition for further time in every instance, for ten minutes, when Dr. Davis, of the Committee on Admissions, read the names of thirty candidates, and also the names of *three* ladies, graduates from Philadelphia, and properly vouched by responsible parties. The third lady is Miss Dr. Brooks, or Dr. Miss, of Youngstown. Miss Brooks is a fair-haired blonde of pleasing appearance. To the intense surprise of every one, the motion to elect passed without a dissenting voice. This was like a clap of thunder from a clear sky. Hereupon Dr. Russel, the venerable old Dr. Russell, protested against the admission of the ladies.

Dr. Reamy then moved a reconsideration of the question in order to make their admission perfectly clear.

Dr. Hamilton also spoke in favor of a reconsideration. 57 in favor, 16 against. The gentlemen doctors were now admitted *en masse*. The second motion to elect the lady doctors now came up.

Dr. Russel now wanted to know whether these ladies were members of a county medical society; if so, he would heartily vote aye, if not he thought it would be more modest in them to withdraw their claims. If there was even any one here who could vouch for them he would vote in their favor. Hereupon an old gentleman presented the requisite voucher and another gentleman stated that Miss Brooks was vice-president of her society.

Dr. Russel said he was glad to see progress. Dr. Hamilton stated that he had a lady student at his lectures all winter and he was not aware that she ever caused him to blush or that any word of his ever caused her to blush. She exercised, on the contrary, a most salutary influence over the conduct of his whole class. The motion now passed unanimously amid loud and prolonged applause. The ladies, the new fellows, were now overwhelmed with congratulations upon all sides.—*Clinic, June.*

